# Limited need for hospital resources among patients brought to hospital by the emergency medical services

**DOI:** 10.1186/s12873-021-00549-6

**Published:** 2021-12-15

**Authors:** Carl Magnusson, Helena Ryge, Filip Scott, Johan Herlitz, Christer Axelsson

**Affiliations:** 1 Institute of Medicine, Department of Molecular and Clinical Medicine, Sahlgrenska Academy, University of Gothenburg, Gothenburg, Sweden; 2 Department of Prehospital Emergency Care, Sahlgrenska University Hospital, Gothenburg, Sweden; 3 Centre for Prehospital Research, Faculty of Caring Science, Work Life and Social Welfare, University of Borås, Borås, Sweden

**Keywords:** Emergency Medical Service, Emergency Department, Hospital Resources

## Abstract

**Background:**

In Sweden, the majority of patients who are transported to hospital by the emergency medical services (EMS) are relatively old and the majority suffer from comorbidity. About half these patients are admitted to a hospital ward and will stay in hospital. However, the other half will only make a visit to the emergency department (ED). The burden on the ED is extensive and many elderly patients have to stay for many hours in the ED.

**Aim:**

To describe the patients who are brought to hospital by the EMS, with particular emphasis on those that were discharged from the ED, and to assess the proportion of these patients who did not require hospital resources, which could mean that they were candidates for primary care (PC).

**Methods:**

An observational analysis of a cohort of patients who were transported to hospital by the EMS in 2016 in the Municipality of Gothenburg.

**Results:**

In all, 5,326 patients were transported to hospital by the EMS of which 52% were discharged directly from the ED. These patients included 37% assessed as not requiring hospital resources. The three most common causes of contact with the EMS in this subset were abdominal pain (15%), back pain (8%) and non-specified disease (7%). Of these patients, 77% had contact with a physician in the ED, whereas 6% had contact with a nurse and 17% left the ED without any contact. Twenty-six per cent were given advice on follow-up in PC.

**Conclusions:**

Among patients who were brought to hospital by the EMS, more than half were discharged directly from the ED. Among these patients, 37% were assessed as not requiring hospital resources. These patients comprised 15% of the overall study cohort and may be candidates for primary care.

## Introduction

One of the key problems in emergency care is the long time many patients have to wait in emergency departments (ED), which is a risk factor for patient safety and low-quality care [1] and which also creates frustration for the patients. This is particularly problematic among the elderly. It has thus been reported that patients aged > 80 years spend a mean of four hours and eight minutes in the ED as compared with patients aged 18-79 years who spend a mean of three hours and 10 minutes in the ED [2]. Simultaneously, an association has been reported between the number of patients assembled in the ED and the risk of death among patients aged > 80 years. An increased risk of death has also been reported if the waiting time in the ED among the elderly is more than eight hours [3].

Many EDs have problems with a high burden of admissions, but, simultaneously, a limited number of beds are available within the hospital. Patients for whom hospitalisation has been determined remain in the ED for hours due to the lack of beds and, at the same time, new admissions are increasing the ED burden still further.

The EMS in Gothenburg, Sweden, handles approximately 60,000 primary missions each year and the median age of these patients is 66 years. Among them, 20% are not conveyed, with advice on self-care or with a referral to PC. Among the remaining patients, 90% are transported directly to the ED and 10% enter a “fast track”, bypassing the ED to a directed investigation or treatment [4]. The latter group includes patients with a suspected hip injury, stroke or myocardial infarction.

In order to relieve the burden of patients on the ED, many EMS systems in Sweden have developed different “chains of care”, with the overall aim of reducing the level of care. These new “chains of care” can result in either advice on self-care or a referral to PC. However, despite these aims, the majority (80%) of patients who are assessed by the EMS are still transported to the ED [4]. However, a significant proportion of the patients who are transported to the ED by the EMS are “low acuity patients” in terms of the severity of disease and they are in fact candidates for a lower level of care [5]. One of the reasons for this finding may be that there are no natural ways from the EMS to primary care (PC) and PC is mostly not available in out-of-office hours.

In order to create secure, safe care for patients, there are two basic requirements: 1) continuity and 2) an appropriate level of care.

The aim of this survey was to describe the patients who are transported to hospital by the EMS, with particular emphasis on those who were directly discharged from the ED. Based on clear definitions, the primary aim was therefore to estimate the proportion of these patients who did not require hospital resources, since a group of this kind may be candidates for a lower level of care.

## Methods

### Catchment area and organisation

The Municipality of Gothenburg has an area of 900 km^2^ and a population of 660,000 people. There are 19 ambulances within the EMS system, of which the majority are available for 24 hours seven days a week. In addition, there are a few vehicles with specific tasks including three single responders for non-emergency cases, one physician-manned unit for cases where a higher competence level is required and one scene-command unit to support more extensive events.

### Competence in the ambulance

According to the Swedish National Board of Health and Welfare, every single ambulance in Sweden should be manned by at least one registered nurse (RN) who is allowed to administer medication. In the study area, each ambulance during the study period was therefore manned by at least one RN and one emergency medical technician (EMT). The majority of the RN:s have a postgraduate education specialising in prehospital emergency care, intensive care or anaesthesia care.

### Decision support tool

The Rapid Emergency Triage and Treatment System (RETTS) is used both in the EDs within the hospital and in the prehospital setting in 95% of the regions in Sweden in order to triage patients. The RETTS consists of measurements of vital signs (VS) and an emergency signs and symptoms (ESS) code which is defined on the basis of cause of contact and symptoms.

When VS and ESS are considered simultaneously, a recommendation on priority level is given by the system.

The priority is divided into five levels, i.e. blue, green, yellow, orange and red.

Blue means that alternatives other than the ED may be appropriate. At the time of the study, this colour was not used when triaging in the prehospital setting. The other four colours mean increasing severity from green to red and the risk of death is expected to increase accordingly.

### Selection of cases

Case selection took place from 1 January until 31 December 31 in 2016. In all, there were 82,000 missions during that year. Among these cases, the first 1,000 were selected each month for the study. After excluding secondary missions (mission without a primary patient assessment), a total of 8,019 patients remained in the study cohort. Among them, 1,307 were excluded due to age < 16 years, patients with incomplete medical records or doublets, patients who died at the scene or for whom a hospital case record was not available.

Among the remaining 6,712 patients, 1,372 were left at the scene and 5,340 patients were transported to hospital (missing information in 14 cases).

This study is based on these 5,326 patients who were transported to hospital, with primary emphasis on the patients who were discharged directly from the ED.

### Case evaluation

The 2,695 patients who were discharged from the ED were divided into two groups: 1) patients who required the use of hospital resources (resources that were considered specific to the hospital and which were not available in PC) and 2) patients who did not require these resources.

The resources that were considered specific to the hospital and were not available in PC were defined as follows.

1. Assessment by a specialist (in neurology, cardiology, infectious diseases, gynaecology etc.);
2. Assessment for the risk of suicide;
3. Extended investigations (computed tomography, ultrasound, lumbar puncture, magnetic resonance imaging, X-ray and scopies);
4. Extended blood sampling (Troponin, d-dimer, NT pro BNP, blood concentrations of drugs);
5. Observation – stayed in the ED for a few hours of observation;
6. Emergency treatment
7. Other (referral from PC)

Group 2 consisted of the patients who were not thought to require hospital resources. Many of these patients had met the physician on call in the department of psychiatry, medicine, orthopaedics and surgery. Many of these patients had undergone a general examination, including basic blood sampling and an ECG recording. Many of them had been given medication, including pain relief and inhalations. Even uncomplicated allergic reactions were considered to belong to a lower level of care, i.e. PC, and were therefore not in need of transport to hospital.

### Statistical methods

The results are presented as frequencies, proportions, the median and 25% and 75% percentiles. Table 1 shows all the patients who were transported to hospital. Tables 2, 3, 4 and 5 show patients who were assessed and treated in the ED but who were then discharged from the ED and were therefore never admitted to a hospital ward.

**Table 1:** Patients assessed by the EMS nurse as requiring hospital resources in relation to whether discharged from the ED

	All	Hospitalised	Discharged from ED
	***n***=5326	***n***=2631	***n***=2695
**Age – years (25th, 75th percentiles)**			
Median	68 (44,83)	76 (59,85)	56 (34,76)
**Gender – n (%)**			
Female	2756 (51.7)	1344 (51.1)	1412 (52.4)
Male	2570 (48.3)	1287 (48.9)	1283 (47.6)
**RETTS ESS code – n (%)**^**1**^ **(160,78,82)**^**2**^			
Abdominal pain	534 (10.3)	206 (8.1)	328 (12.6)
Chest pain	512 (9.9)	256 (10.0)	256 (9.8)
Dyspnea	454 (8.8)	334 (13.1)	120 (4.6)
Trauma, head	323 (6.3)	81 (3.2)	242 (9.3)
Non-specific disease	349 (6.8)	221 (8.7)	128 (4.9)
Intoxication	251 (4.9)	130 (5.1)	121 (4.6)
Vertigo	191 (3.7)	94 (3.7)	97 (3.7)
Trauma, hip/leg	186 (3.6)	136 (5.3)	50 (1.9)
Infection	137 (2.7)	109 (4.3)	28 (1.1)
Back pain	130 (2.5)	30 (1.2)	100 (3.8)
**Triage colour – n (%)**			
Red	596 (11.6)	487 (19.1)	109 (4.2)
Orange	1853 (35.9)	992 (39.0)	861 (33.0)
Yellow	2247 (43.6)	928 (36.5)	1319 (50.5)
Green	459 (8.9)	137 (5.4)	322 (12.3)
Triage colour missed	171 (3.2)	87 (3.3)	84 (3.1)
**Time of day and night – n (%)**			
8-16	2424 (45.5)	1286 (48.9)	1138 (42.2)
16-22	1523 (28.6)	749 (28.5)	774 (28.7)
22-08	1379 (25.9)	596 (22.7)	783 (29.1)

^1^Ten most common ESS codes

^2^Number of missions with missing ESS code

**Table 2:** Patients in need of emergency department resources

		GENDER		AGE	
Type of resources^**1**^ – n (%)	All	Female	Male	< 56 years	≥ 56 years
**OR (95% Cl)**^**2**^	**1689**	**899**	**790**	**803**	**886**
Specialist evaluation	220 (13.0)	124 (13.8)	0.87 (0.65-1.15)	111 (13.8)	0.88 (0.66-1.16)
Suicide evaluation	55 (3.3)	36 (4.0)	0.59 (0.34-1.04)	45 (5.6)	0.19 (0.10-0.38)
Extended evaluation^3^	852 (50.4)	438 (48.7)	1.16 (0.96-1.40)	396 (49.3)	1.09 (0.90-1.32)
Blood sampling	438 (25.9)	243 (27.0)	0.89 (0.71-1.10)	172 (21.4)	1.57 (1.26-1.96)
Observation	65 (3.8)	33 (3.7)	1.11 (0.68-1.82)	48 (6.0)	0.31 (0.18-0.54)
Emergency care	39 (2.3)	16 (1.8)	1.66 (0.87-3.16)	18 (2.2)	1.06 (0.56-2.00)
Other^4^	20 (1.2)	9 (1.0)	1.40 (0.58-3.39)	13 (1.6)	0.48 (0.19-1.22)

^1^Each patient belongs to only one category in decreasing order

^2^OR: odds ratio, 95% confidence interval

^3^Computed tomography, ultrasound, lumbar puncture, magnetic resonance imaging, X-ray, scopies

^4^Patients with referral from primary care; ED physician want to admit but the patient opposes it.

**Table 3:** Patients not in need of hospital resources

		GENDER		AGE	
Type of evaluation^**1**^ – n (%)	All	Female	Male	< 56 years	≥ 56 years
**OR (95% Cl)**^**2**^	**1006**	**513**	**493**	**522**	**484**
Contact with physician, treatment with or prescription of drugs	332 (33.0)	178 (34.7)	0.86 (0.66-1.11)	155 (29.7)	1.37 (1.05-1.78)
Contact with a physician	446 (44.3)	225 (43.9)	1.04 (0.81-1.33)	214 (41.0)	1.33 (1.03-1.70)
Contact with a nurse	56 (5.6)	24 (4.7)	1.41 (0.82-2.44)	36 (6.9)	0.58 (0.33-1.02)
Patient left the scene	172 (17.1)	86 (16.8)	1.05 (0.76-1.46)	117 (22.4)	0.44 (0.31-0.63)
Follow-up at a lower level of care^3^	259 (25.7)	122 (23.8)	1.23 (0.93-1.64)	120 (23.0)	1.35 (1.02-1.79)

^1^Each patient belongs to only one category in decreasing order

^2^OR: odds ratio, 95% confidence interval

^3^Patients for whom the emergency department physician, nurse, wants a follow-up in primary care, alcohol-dependence clinic, reception

**Table 4:** Patient characteristics and prehospital assessment in relation to need for hospital resources among patients discharged from the ED

Need for hospital resources		No	Yes	OR (univariate)^**1**^
		n=1006	n=1689	
Age (years)^2^	16-33	254 (25.2)	392 (23.2)	
	34-55	268 (26.6)	411 (24.3)	0.99 (0.80-1.24, p=0.955)
	56-75	251 (25.0)	434 (25.7)	1.12 (0.90-1.40, p=0.315)
	>75	233 (23.2)	452 (26.8)	1.26 (1.01-1.57, p=0.045)
Gender	Female	513 (51.0)	899 (53.2)	
	Male	493 (49.0)	790 (46.8)	0.91 (0.78-1.07, p=0.262)
Dispatch (17)^3^	Prio 1	432 (43.3)	859 (51.1)	
	Prio 2	509 (51.0)	763 (45.4)	0.75 (0.64-0.89, p=0.001)
	Prio 3	57 (5.7)	58 (3.5)	0.51 (0.35-0.75, p=0.001)
Triage colour (84)	Red	14 (1.4)	95 (5.8)	
	Orange	234 (23.9)	627 (38.4)	0.39 (0.21-0.68, p=0.002)
	Yellow	549 (56.1)	770 (47.2)	0.21 (0.11-0.35, p<0.001)
	Green	181 (18.5)	141 (8.6)	0.11 (0.06-0.20, p<0.001)
Previous history	Healthy	181 (18.0)	329 (19.5)	
	1	208 (20.7)	331 (19.6)	0.88 (0.68-1.12, p=0.299)
	2	187 (18.6)	269 (15.9)	0.79 (0.61-1.03, p=0.078)
	≥3	430 (42.7)	760 (45.0)	0.97 (0.78-1.21, p=0.800)
Time of day	08 - 16	392 (39.0)	746 (44.2)	
	16-22	316 (31.4)	458 (27.1)	0.76 (0.63-0.92, p=0.005)
	22-08	298 (29.6)	485 (28.7)	0.86 (0.71-1.03, p=0.105)
Alcohol/drugs	Yes	128 (12.7)	188 (11.1)	0.86 (0.68-1.09, p=0.214)
Medication	Yes	215 (21.4)	458 (27.1)	1.37 (1.14-1.65, p=0.001)

^1^OR: odds ratio, 95% confidence interval

^2^Age, categorised by quartiles

^3^Number of patients with missing information

**Table 5:** Prehospital assessment according to RETTS in relation to need for hospital resources

Need for hospital resources	No	Yes	OR (univariate)^**1**^
	***n***=1006	***n***=1689	
**ESS code**^**2**^ **(82)**^**3**^			
Abdominal pain	150 (15.3)	178 (10.9)	0.68 (0.54-0.85, p=0.001)
Trauma, extremities/thorax^4^	59 (6.0)	237 (14.5)	2.65 (1.98-3.59, p<0.001)
Chest pain	42 (4.3)	214 (13.1)	3.36 (2.42-4.79, p<0.001)
Trauma, head	64 (6.5)	178 (10.9)	1.75 (1.31-2.37, p<0.001)
Intoxication	57 (5.8)	65 (4.0)	0.67 (0.47-0.97, p=0.032)
Dyspnea	53 (5.4)	67 (4.1)	0.75 (0.52-1.08, p=0.122)
Non-specific disease	71 (7.3)	57 (3.5)	0.46 (0.32-0.66, p<0.001)
Back pain	75 (7.7)	30 (1.8)	0.23 (0.14-0.34, p<0.001)
Dizziness	55 (5.6)	42 (2.6)	0.44 (0.29-0.67, p<0.001)
Seizures	19 (1.9)	68 (4.2)	2.19 (1.34-3.77, p=0.003)
Psychiatric abuse	47 (4.8)	36 (2.2)	0.45 (0.29-0.69, p<0.001)
Syncope	38 (3.9)	43 (2.6)	0.67 (0.43-1.05, p=0.076)
Trauma, alarm	8 (0.8)	68 (4.2)	5.27 (2.68-11.94, p<0.001)
Headache	33 (3.4)	43 (2.6)	0.77 (0.49-1.24, p=0.278)
Fever, infection	24 (2.5)	34 (2.1)	0.85 (0.50-1.45, p=0.534)
Other^5^	185 (18.9)	274 (16.8)	0.86 (0.70-1.06, p=0.167)

^1^OR: odds ratio, 95% confidence interval

^2^The 15 most frequent ESS codes

^3^Number of patients with missing information

^4^Includes trauma to extremity, hip, pelvis and thorax

^5^Includes ESS codes, atrial fibrillation, neurological deficit, pain/swollen extremity, urological problems, allergy

Table 1 shows age, gender, the 10 most common RETTS ESS codes among all cases, triage colours and the time of day of admission to the ED.

In Tables 2 and 3, patients who did and did not require specific hospital resources are presented as overall numbers and in subsets according to the type of resources used (Table 2) and various actions if no hospital resources were required (Table 3). In both tables, the results are also presented according to gender and age.

In the statistical analyses, odds ratios (OR) univariate and 95% confidence intervals (CI) and p-values were used. All p-values are two-sided and p-values below 0.05 were considered significant. In Tables 2 and 3, women and age below the median comprised the references in the calculation of OR and 95% CI.

In Tables 4 and 5, the two groups assessed as needing and not needing hospital resources have been compared. In Table 4, patients have been divided into quartiles among all patients. Age 16-33 years, women, dispatch priority 1, triage colour red, no previous disease and time of day 08-16 comprised the references. Time of day was categorised in relation to when PC was available (08-16).

In Table 5, patients needing and not needing hospital resources are compared regarding the 15 most common ESS codes for all patients who were discharged from the ED.

SPSS version 25 (IBM Corp, Armonk, NY) was used for data processing and statistical analyses were performed in R-studio version 1.2.5 (RStudio Inc., Boston, MA).

## Results

**Fig. 1:**
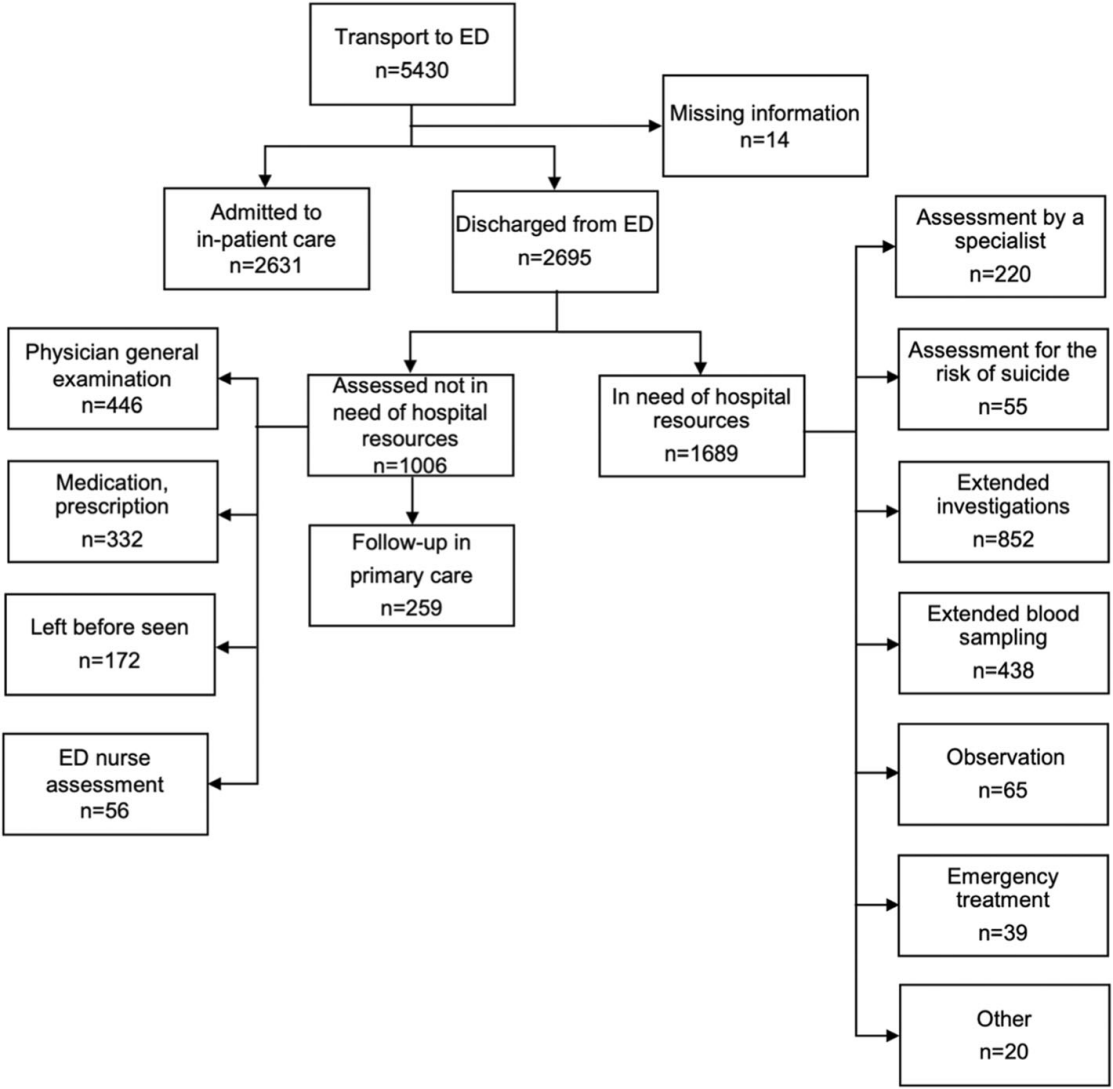
Distribution of patients assessed and transported to the ED. ED:Emergency department; Extended investigations: computed tomography, ultrasound, lumbar puncture, magnetic resonance imaging, X-ray and scopies; Extended blood sampling: Troponin, d-dimer, NT pro BNP, blood concentrations of drugs; Other: Referral from primary care, in-patient care refusal

In the study population, 5,326 patients were transported to hospital by the EMS. Among these patients, 2,631 (49.4%) were hospitalised and 2,695 (51.6%) were discharged from the ED. Among the 2,695 patients who were discharged from the ED, we found that 1,006 (37.4%) did not use any of the resources which by definition were unique to the hospital (Figure 1).

### A comparison between patients who were and were not hospitalised (Table 1)

Patients who were hospitalised had a median age which was 20 years higher than those who were not hospitalised, whereas the distribution of gender did not differ.

Among all patients, the three most common ESS codes in order of frequency were: 1) abdominal pain (10.3 %); 2) chest pain (9.9%) and 3) dyspnea (8.8%). Among the patients who were hospitalised, the three most common ESS codes were: 1) dyspnea; 2) chest pain and 3) non-specific disease, whereas, among the patients who were discharged from the ED, the three most common ESS codes were: 1) abdominal pain, 2) chest pain and 3) head trauma.

The proportion of patients who were triaged to red by the EMS at the scene was 19.1% among patients who were hospitalised versus 4.2% among patients who were discharged from hospital.

### Patients in need of hospital resources (Table 2)

Among the patients who were assessed as being in need of hospital resources, 50.4% required an extended evaluation and 25.9 % required extended blood sampling. A suicide evaluation was more common among younger patients, whereas extended blood sampling was more common among the elderly.

### Patients not in need of hospital resources (Table 3)

Among the patients who were assessed as not being in need of hospital resources, 44.3% had contact with a physician and 33.0% had contact with a physician and were given medication either directly or on prescription. Advice on follow-up in PC was given to 26% of the patients. The elderly more frequently had contact with a physician, were more frequently given medication and were more frequently advised to go for a follow-up in PC.

### Characteristics of patients who required hospital resources (Table 4)

Patients aged > 75 years more frequently required hospital resources. Patients who received priority 2 and 3 at dispatch, as well as patients who were triaged yellow and green by the EMS nurse, less frequently required hospital resources. Patients who sought care between 16.00-22.00 less frequently required hospital resources as compared with those who sought care during the daytime. Finally, patients who were given medication by the EMS crew more frequently required hospital resources.

### Prehospital assessment according to RETTS among patients who did and did not require hospital resources (Table 5)

Among patients who did not require hospital resources, abdominal pain, back pain and non-specified sickness were the most common ESS codes in the prehospital assessment. Among patients who required hospital resources, trauma (all subsets added) and chest pain were the most common ESS codes in the prehospital assessment.

## Discussion

The main findings in this study were that, among patients who were brought to hospital by the EMS, 52% were directly discharged from the ED and, among these patients, a substantial percentage (37%) did not require hospital resources. Patients who did not require hospital resources were younger than those who did require these resources and they were given a lower priority in the dispatch centre, as well as by the EMS nurse. Among those who did not require hospital resources, the most common cause of contact with the EMS was abdominal pain (15%).

The most common ESS codes among all the patients who were transported to hospital were abdominal pain, chest pain and dyspnea. However, among these subsets, the majority were admitted to a hospital ward among those with dyspnea and chest pain, whereas, on the other hand, among patients with abdominal pain, the majority were discharged from the ED. In overall terms, patients who were admitted to a hospital ward were 20 years older than those who were discharged from the ED.

Among patients who were admitted to a hospital ward, 56% were triaged to red or orange (the highest priority), whereas, among patients who were discharged from the ED, 81% were triaged to green or yellow (the lowest priority). As a result, prehospital triage predicted the subsequent outcome to some extent, particularly among patients who were given a low priority.

In overall terms, 49% of all the patients who were transported to hospital by the EMS were hospitalised. This finding differs from a US study in which only 20% of patients with acute symptoms were hospitalised [6]. However, that study used a different triage system.

Previous experience has suggested that 16% of patients assessed by the EMS are candidates for PC [7]. However, in the present study, 20% of patients were left at the scene. In addition, 1,006 patients (37% of those who were discharged from the ED) were potential candidates for PC. This subset comprised 15% of all primary missions in the survey (n=6,712). According to the experience acquired from the present study, 20% + 15% = 35% of primary missions in prehospital emergency care are potential candidates for PC. A previous study from Sweden suggested that 85% of EMS missions did not have a life-threatening or an acute condition [8]. Others have suggested that between 20-40% of patients admitted to the ED can be handled by PC [9]. In fact, 26% of the patients in the present study cohort who were discharged from the ED and not assessed as requiring hospital resources were advised to seek a lower level of care.

The majority of patients who were admitted between 08-16 required hospital resources, whereas the opposite was found among patients admitted between 16-22. One of the reasons behind this finding may be that a large number of out-patient clinics close at 16.00. This hypothesis was supported by Fry et al. who reported that many patients explained their admission to the ED by the fact that the out-patient clinic was closed [10].

It is reasonable to assume that a number of patients seek emergency care at the ED due to limited access to PC. This is supported by Tinkler et al., who reported that the median delay to see a doctor in PC was 22 days [11]. It seems as though many patients who visit the ED are not aware of any other alternative [12].

A future alternative introduced in the UK is the “nurse practitioner” (NP) who has an extended education. An NP has increased responsibility as compared with an RN and is allowed to prescribe medication, among other things [13].

It is possible to discuss whether patients’ previous history should be considered in prehospital triage. Previous studies have suggested that comorbidity increases the likelihood that admissions to the ED are the appropriate level of care and that the presence of comorbidity is associated with an increased need for hospital resources [7, 14]. This was not confirmed in the present study. It is possible to argue that the vast majority of patients in our survey suffered from some previous disease (87%), which may have created difficulties in addressing this question.

The three most common causes of contact with the EMS according to RETTS and the ESS code among the patients who were assessed as not requiring hospital resources were abdominal pain, back pain and non-specific disease. However, on the other hand, many patients were given these ESS codes, admitted to a hospital ward and thus actually required the hospital resources. The EMS nurses in Sweden thus need improved decision-support tools in the prehospital setting to be able to assess patients with these symptoms to the optimal level of care.

### Limitation

The survey was performed in 2016 and several years have passed since then. Some minor changes in the EMS system have taken place. For example, mobile out-patient care teams have been created. However, the routines associated with the prehospital assessment by the EMS nurse have remained the same.

The criteria for using hospital resources can be discussed. In fact, some of the blood samples, such as Troponin, may have already been assessed in PC.

## Conclusions

Among patients who were brought to hospital by the EMS, more than half were directly discharged from the ED. Among these patients, 37% were assessed as not requiring hospital resources. These patients comprised 15% of the overall study cohort and may be candidates for primary care.

## Data Availability

Data sets are available from the corresponding author on reasonable request.

## Abbreviations

ECG: Electrocardiogram
ED: Emergency department
EMS: Emergency medical services
ESS: Emergency signs and symptoms
NP: Nurse practitioner
NT-pro BNP: N-terminal pro b-type natriuretic peptide
PC: Primary care
RETTS: Rapid emergency triage and treatment system
RN: Registered nurse
VS: Vital signs
UK: United Kingdom
